# Coronal alignment in normal individuals and moderate scoliosis: Normative values, variation with age and comparison with sagittal alignment

**DOI:** 10.1016/j.bas.2024.103917

**Published:** 2024-10-11

**Authors:** Ahmed Gharbi, Ibrahim Obeid, Daniel Larrieu, Louis Boissière, Maxime Huneidi, Pablo Lamotte-Paulet, Mekki Tamir, Carlos Aleman, Yann Philippe Charles

**Affiliations:** a Department of Spine Surgery, Strasbourg University Hospital, Strasbourg, France; b Spine Surgery Unit 1, Bordeaux University Hospital, Bordeaux, France

**Keywords:** Coronal alignment, Normative values, Sagittal alignment, Radiographic parameters, Age, Mild scoliosis

## Abstract

**Introduction:**

Global coronal alignment is mainly assessed by C7 plumbline and central sacral vertical line (CSVL), pelvic obliquity and shoulder alignment. A detailed analysis is mandatory when treating spinal deformity. It remains unclear to what extent mild scoliosis influences global coronal alignment.

**Research question:**

The objective was to define a comprehensive set of coronal alignment parameters and to investigate differences between individuals without spinal deformity and with mild scoliosis. The relationship between coronal and sagittal alignment and the influence of age were studied.

**Methods:**

Radiographs of 236 normal individuals (Group N) and 140 patients with scoliosis <35° (Group S) were prospectively collected. Coronal parameters were femoral head distance and angle, pelvic obliquity, Maloney angle, L4 and L5 inclinations, coronal T1 pelvic angle, C7-CSVL and odontoid CSVL offset, coracoid distance and angle. Sagittal cervical, spinopelvic, thoracolumbar and global parameters were measured.

**Results:**

There was no significant difference between groups N and S for coronal parameters, except for L4 and L5 inclinations with a mean difference of 3,3° (p < 0,001). Global coronal alignment kept constant throughout age groups in N and S groups. Sagittal parameters varied with age: C2-C7 lordosis (p < 0,001), T1-T12 kyphosis (p < 0,001), pelvic incidence (p < 0,001). There was no correlation between global coronal and sagittal alignment: R-values ranging from −0.2 to 0.2.

**Conclusion:**

Global coronal parameters were comparable in normal individuals and in scoliosis <35°. Coronal plane parameters were not influenced by age. Sagittal plane parameters varied significantly with age. There was no direct link between coronal et sagittal alignment.

## Highlights

• This study provides a comprehensive description and normative values for coronal alignment parameters.
• Global coronal alignment is similar between normal subjects without spinal deformity and in patients with scoliosis <35°.
• Coronal parameters in normal subjects and mild scoliosis don’t vary with age, whereas variations exist in the sagittal plane.
• There is no correlation between the coronal and the sagittal alignment parameters in normal subjects and mild scoliosis.

## 1 Introduction

Analyzing coronal alignment is crucial in spinal deformity. Through the last decade, our understanding of sagittal alignment improved, and the number of clinical applications increased. On the other hand, coronal alignment analysis is often limited to C7 plumbline (C7PL) and Cobb angle assessment when dealing with spinal deformity. Bao et al. (2016) classified adult scoliosis into 3 categories: distance between C7PL and central sacral vertical line (CSVL) < 3 cm, C7PL shift >3 cm to the curve convexity or concavity. Obeid et al. (2019) described a comprehensive coronal malalignment algorithm for adult spinal deformity (ASD) and scoliosis that distinguishes concave and convex coronal malalignment patterns and account for curve location and flexibility.

In clinical practice, proper coronal alignment setting can be challenging in ASD surgery. Ploumis et al. (2015) showed that 16% of preoperatively malaligned patients kept malaligned postoperatively and that coronal alignment could worsen in some patients. Buell et al. (2021) demonstrated that instrumentation length and fusion to the pelvis influences the risk for postoperative malalignment. As pre- and postoperative patient reported outcome measures are linked to coronal alignment (Buell et al., 2021; Shen et al., 2024), it is important to have a thorough understanding of normal coronal alignment and pathologic malalignment in spinal deformity. Shen et al. (2024) investigated the relationship between the orbit, the odontoid and C7 related to L5. Zuckermann et al. (Zuckerman et al., 2021) defined normative values the odontoid related to the CSVL, and the influence of pelvic obliquity was further investigated (Zuckerman et al., 2024). It seems obvious that severe spinal deformity can affect global coronal alignment. However, it remains unclear to what extent mild scoliosis influence global alignment. Furthermore, it remains unclear if age and sagittal alignment parameters are related to coronal alignment.

The purpose of this cross-sectional study was to describe a comprehensive set of normative coronal alignment parameters in asymptomatic subjects and to compare those to patients with mild scoliosis. The influence of age was studied. The relationship between coronal and sagittal cervical, thoracolumbar, pelvic, and global alignment parameters was further investigated.

## 2 Materials and methods

Institutional review board approval (FC/2019-91) was obtained. Coronal and sagittal full EOS spine radiographs were performed in two spine surgery centers. Radiographs of healthy individuals and of patients who were screened for scoliosis were prospectively collected from December 2022 to October 2023 and analyzed retrospectively. Exclusion criteria were spinal or pelvic fractures, tumors, infection and neuromuscular disorders, previous spine surgery other than microdiscectomy and severe degenerative changes or osteoporosis leading to thoracolumbar deformity, previous total hip arthroplasty or hemiarthroplasty and spinal deformity such as scoliosis of >35°. Mild scoliosis <35° have been included. Two groups were defined: a normal N-group of asymptomatic individuals without coronal curve and a scoliosis S-group with moderate Cobb angle between 15° and 35°.

**Fig. 1:**
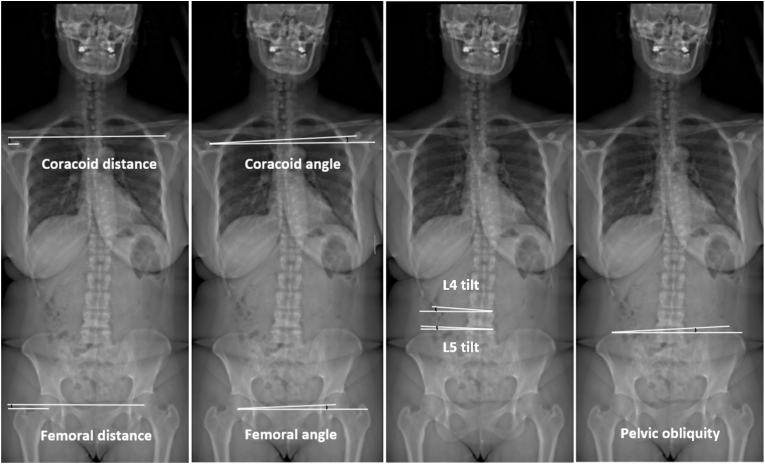
Coronal parameters assessing pelvic, lumbar and shoulder alignment: coracoid distance, coracoid angle, femoral head distance, femoral head angle, L4 and L5 tilt, pelvic obliquity.

**Fig. 2:**
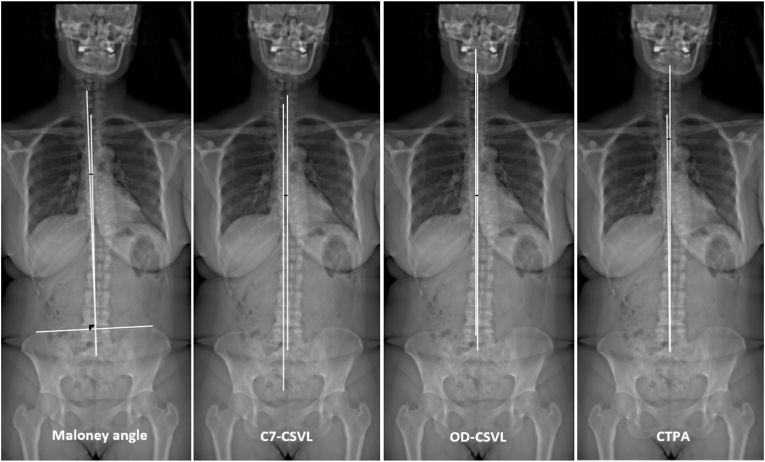
Parameters assessing global coronal alignment: Maloney angle, C7 central sacral vertical line (C7-CSVL), odontoid central sacral vertical line (OD-CSVL), coronal T1 pelvic angle (CTPA).

Five trained operators reconstructed radiographic landmarks between C1 and femoral heads in the coronal plane and between the cranial auditory meatus and femoral heads in the sagittal plane using KEOPS software (SMAIO, Lyon, France). Two senior spine surgeons reviewed all reconstructions to minimize inter-rater errors. This method is reliable and superior to manual measurements (Maillot et al., 2015). Coronal parameters were measured and assessed pelvic, lumbar and shoulder alignment (Fig. 1) as well as global coronal alignment (Fig. 2):

- *Femoral head distance* was measured as the distance (mm) between horizontal lines passing through femoral head centroids.
- *Femoral head angle* was measured between the horizontal line and the line joining both femoral head centroids.
- *Pelvic obliquity* was measured as the angle between the axis tangent to both iliac crests and the horizontal line.
- *L4 and L5 tilt* were measured as the inclination angle of the cranial endplate of respective vertebrae related to the horizontal axis.
- *Coracoid distance* was measured as the distance (mm) between horizontal lines passing through coracoid centroids.
- *Coracoid angle* was measured between the horizontal line and the line joining both coracoid centroids.
- *Maloney angle* represented the angle between the axis joining the center of T1 to the center of the sacrum and the axis perpendicular to the tangent to both iliac crests passing trough the center of the sacrum (Fann, 2003).
- *Coronal T1 pelvic angle (CTPA)* was defined as the angle between a vertical line and the line joining the midpoint of the S1 endplate and the T1 centroid (Zhang et al., 2020).
- *C7 central sacral vertical line (C7-CSVL)* was the distance between C7 plumbline and the midpoint of the S1 endplate (mm).
- *Odontoid central sacral vertical line (OD-CSVL)* was the distance between the odontoid plumbline and the midpoint of the S1 endplate (mm) (Zuckerman et al., 2021).

Sagittal parameters were previously described (Sardar et al., 2023; Charles et al., 2022a) and grouped by type.

- *Cervical parameters* were McGregor slope, McGregor-C2 angle, cervical lordosis (CL) C1-C2, CL C2-C7, C7 and T1 slope.
- *Thoracolumbar parameters* were thoracic kyphosis (TK) T1-T12, TK T5-T12 and lumbar lordosis (LL) L1-S1.
- *Pelvic parameters* were pelvic incidence (PI), pelvic tilt (PT), sacral slope (SS) and PI-LL.
- *Global alignment parameters* were sagittal vertical axis (SVA) of C7 and C2, T1 pelvic angle (TPA), global tilt (GT), spino-sacral angle (SSA), T1-tilt, T9-tilt, and the odontoid hip axis (OD-HA).

Statistical evaluation was performed with RStudio software (2023.12.0 Build 369, R Foundation for Statistical Computing, Vienna, Austria). Continuous variables were expressed as means, standard deviation and 95% credible intervals. Normal distribution was tested using a Shapiro-Wilk test. A student t-test was used for normally distributed parameters and a Wilcoxon test for non-normally distributed data when comparing respective parameters between N- and S-groups. An ANOVA was used for comparison between age groups. Pearson correlation coefficients were calculated between coronal and sagittal parameters. A p-value <0.05 was considered statistically significant.

## 3 Results

A total of 376 individuals, 209 females and 167 males were included. Ages ranged from 6 to 89 years. The distribution by age was <20 years n = 128, 20–50 years n = 114, >50 years n = 134. The N-group 236 included individuals and the S-group included 140 patients with mild scoliosis.

### 3.1 Coronal parameters

Table 1 displays coronal parameters for N- and S-groups. The mean Cobb angle in the S-group was 20.0°. A mean difference of 3.3° between groups was found for L4 and L5 tilt respectively (p < 0.0001). Although statistically significant, differences were minor for CTPA 0.3° (p = 0.004), C7-CSVL 1.9 mm (p = 0.007), OD-CSVL 0.2 mm (p = 0.015), and below the threshold of radiographic measurement accuracy. No significant differences between N- and S-groups were found for coronal pelvic and shoulder parameters: femoral head angle and distance, Maloney angle, coracoid angle and distance.

**Table 1:** Coronal parameters comparing normal (N) and scoliosis (S) groups.

Variable	N-group		S-group		
	Mean ± SD	95%CI	Mean ± SD	95%CI	p
Femoral head angle (°)	1.6 ± 1.2	[1.4; 1.7]	1.9 ± 1.6	[1.6; 2.2]	0.339
Femoral head distance (mm)	4.7 ± 3.4	[4.2; 5.1]	5.5 ± 4.6	[4.7; 6.3]	0.304
Pelvic obliquity (°)	1.7 ± 1.3	[1.5; 1.9]	2.1 ± 1.7	[1.8; 2.4]	0.054
Maloney angle (°)	3.5 ± 11.2	[2.1; 5]	3 ± 7.5	[1.7; 4.3]	0.105
L4 tilt (°)	3.0 ± 2.3	[2.7; 3.3]	6.3 ± 5	[5.5; 7.1]	0.000
L5 tilt (°)	3.0 ± 2.3	[2.7; 3.3]	6.3 ± 5	[5.5; 7.1]	0.000
Coronal T1 pelvic angle (°)	1.1 ± 1.0	[1.0; 1.3]	1.4 ± 1.1	[1.2; 1.6]	0.004
C7 CSVL (mm)	8.3 ± 7.5	[7.4; 9.3]	10.2 ± 7.8	[8.9; 11.5]	0.007
Odontoid CSVL angle (°)	1.0 ± 0.9	[0.9; 1.1]	1.2 ± 0.9	[1.1; 1.4]	0.015
Coracoid angle (°)	1.8 ± 1.4	[1.6; 2.0]	2.0 ± 1.6	[1.7; 2.3]	0.298
Coracoid distance (mm)	7.1 ± 5.7	[6.4; 7.8]	8.0 ± 6.5	[6.8; 9]	0.348

### 3.2 Variation of coronal parameters with age

Table 2, Table 3 show the variation of coronal parameters per age category <20 years, 20–50 years and >50 years in the N-group and in the S-group respectively. There was no significant difference between the three age categories in either group, except for OD-CSVL with an increase of 0.5 mm increase in the >50-year category (p = 0.017), below the threshold of radiographic measurement accuracy.

**Table 2:** Comparison of coronal parameters (mean ± SD) per age in the normal (N) group.

Variable	<20 years	20–50 years	>50 years	p
Femoral head angle (°)	1.7 ± 1.2	1.5 ± 1.2	1.6 ± 1.0	0.497
Femoral head distance (mm)	4.6 ± 3.3	4.5 ± 3.8	5.0 ± 3.2	0.623
Pelvic obliquity (°)	1.8 ± 1.3	1.7 ± 1.4	1.6 ± 1.1	0.476
Maloney angle (°)	2.2 ± 1.7	3.2 ± 9.9	5.2 ± 16.3	0.214
L4 tilt (°)	3.0 ± 2.3	2.8 ± 2.3	3.2 ± 2.3	0.588
L5 tilt (°)	3.0 ± 2.3	2.8 ± 2.3	3.2 ± 2.3	0.588
Coronal T1 pelvic angle (°)	1.1 ± 1.0	1.0 ± 0.8	1.3 ± 1.1	0.161
C7 CSVL (mm)	7.5 ± 7.1	7.6 ± 6.4	9.7 ± 8.7	0.108
Odontoid CSVL angle (°)	0.9 ± 0.9	0.9 ± 0.7	1.2 ± 1.1	0.017
Coracoid angle (°)	1.7 ± 1.3	1.8 ± 1.2	2.0 ± 1.6	0.396
Coracoid distance (mm)	6.4 ± 5.3	7.1 ± 5	7.7 ± 6.7	0.330

**Table 3:** Comparison of coronal parameters (mean ± SD) per age in the scoliosis (S) group.

Variable	<20 years	20–50 years	>50 years	p
Cobb angle (°)	20.5 ± 3.7	20.4 ± 4.8	19.3 ± 4.3	0.327
Femoral head angle (°)	2.3 ± 1.9	1.6 ± 1.4	1.7 ± 1.4	0.085
Femoral head distance (mm)	6.1 ± 4.9	5.1 ± 4.5	5.3 ± 4.6	0.560
Pelvic obliquity (°)	2.4 ± 2.1	1.9 ± 1.3	2.1 ± 1.5	0.356
Maloney angle (°)	2.5 ± 2.0	2.3 ± 1.5	4.0 ± 1.2	0.496
L4 tilt (°)	6.4 ± 4.6	5 ± 4.5	7.1 ± 5.3	0.150
L5 tilt (°)	6.4 ± 4.6	5 ± 4.5	7.1 ± 5.3	0.150
Coronal T1 pelvic angle (°)	1.3 ± 0.9	1.3 ± 1.2	1.6 ± 1.2	0.305
C7 CSVL (mm)	9.0 ± 6.0	10 ± 8.9	11.5 ± 8.5	0.252
Odontoid CSVL angle (°)	1.2 ± 0.8	1.1 ± 1.0	1.3 ± 1.0	0.445
Coracoid angle (°)	1.8 ± 1.6	2.0 ± 1.7	2.2 ± 1.5	0.301
Coracoid distance (mm)	6.6 ± 6.0	8.6 ± 8.1	8.6 ± 5.8	0.234

### 3.3 Sagittal parameters

Table 4 displays sagittal parameters for N- and S-groups. There was no significant difference between the two groups for each of the cervical, thoracolumbar, pelvic, or global alignment parameters.

**Table 4:** Sagittal parameters (N) and scoliosis (S) groups.

Variable	N-group		S-group		
	Mean ± SD	%95CI	Mean ± SD	%95CI	p
McGregor slope (°)	1.7 ± 8.9	[0.5; 2.8]	2.9 ± 17.6	[0.1; 5.9]	0.895
McGregor C2 angle (°)	19.2 ± 10.9	[17.8; 20.6]	17.3 ± 11.5	[15.3; 19.2]	0.052
Cervical lordosis C1-C2 (°)	29.5 ± 8.3	[28.5; 30.6]	28.8 ± 9.7	[27.2; 30.4]	0.460
Cervical lordosis C2-C7 (°)	25.1 ± 14.1	[23.2; 26.9]	27.4 ± 16.1	[24.7; 30.1]	0.386
C7 slope (°)	22.5 ± 11.2	[21.0; 23.9]	21.1 ± 12.4	[19.0; 23.2]	0.159
T1 slope (°)	26.5 ± 11.1	[25.0; 27.9]	24.0 ± 11.9	[22.1; 26.1]	0.076
Thoracic kyphosis T1-T12 (°)	45.6 ± 14.3	[43.7; 47.4]	44.0 ± 14.3	[41.6; 46.4]	0.295
Thoracic kyphosis T5-T12 (°)	34.0 ± 12.4	[32.4; 35.6]	36.0 ± 13.7	[33.2; 37.8]	0.199
Lumbar lordosis L1-S1 (°)	55.5 ± 13.1	[53.8; 57.1]	56.7 ± 13.7	[54.4; 59.0]	0.381
Pelvic incidence (°)	51.9 ± 12.6	[50.3; 53.5]	50.3 ± 13.2	[48.1; 52.5]	0.319
Sacral slope (°)	38.1 ± 10.2	[36.8; 39.4]	38.4 ± 9.2	[36.8; 39.9]	0.544
Pelvic tilt (°)	13.9 ± 8.9	[12.7; 15.0]	11.9 ± 11.0	[10.1; 13.8]	0.078
PI-LL L1-S1 (°)	−3.5 ± 11.9	[-5.1; −2.0]	−6.5 ± 15.6	[-9.1; −3.9]	0.097
SVA C7 (mm)	15.1 ± 35.8	[10.5; 19.6]	15.1 ± 36.6	[9.0; 21.2]	0.886
SVA C2 (mm)	34.2 ± 41.2	[29.0; 39.5]	31.8 ± 41.7	[24.8; 38.7]	0.676
T1 pelvic angle (°)	11.3 ± 7.4	[10.4; 12.3]	10.9 ± 8.4	[9.5; 12.3]	0.325
Global tilt (°)	13.8 ± 8.8	[12.7; 15.0]	11.8 ± 10.2	[10.1; 13.5]	0.055
Spino-sacral angle (°)	128.1 ± 10.6	[126.7; 129.4]	128.3 ± 10.5	[126.5; 130]	0.673
Odontoid hip axis (°)	0.6 ± 3.2	[0.2; 1.0]	0.6 ± 3.0	[0.1; 1.1]	0.821
T1 tilt (°)	3.5 ± 3.5	[3.1; 4.0]	3.0 ± 3.4	[2.4; 3.6]	0.163
T9 tilt (°)	9.8 ± 4.1	[9.3; 10.4]	9.2 ± 4.8	[8.4; 10.0]	0.237

### 3.4 Variation of sagittal parameters with age

In the sagittal plane, there were significant difference between age categories for most of the studied parameters. This difference applied similarly N- and S-groups. Table 5, Table 6 show variations of sagittal parameters depending on age categories. Among cervical parameters, McGregor slope and the McGregor-C2 angle, CL C2-C7, C7 and T1 slope increased with age, whereas CL C1-C2 kept stable. Among thoracolumbar parameters, TK T1-T12 and T5-T12 increased with age, whereas LL L1-S1 increased from children to adulthood, and then decreased with age. Among pelvic parameters, PI and PT increased, whereas SS decreased with age. Among global alignment parameters, SVA C2 and C7, TPA, GT and T9 tilt increased, whereas SSA, T1 tilt and OD-HA decreased in both groups.

**Table 5:** Comparison of sagittal parameters (mean ± SD) per age in the normal (N) group.

Variable	<20 years	20–50 years	>50 years	p
McGregor slope (°)	−1.4 ± 9.5	1.2 ± 7.6	5.1 ± 8.4	0.000
McGregor C2 angle (°)	15.1 ± 11.2	20.2 ± 10.5	22.2 ± 10.0	0.000
Cervical lordosis C1-C2 (°)	28.9 ± 9.3	29.9 ± 7.9	29.8 ± 7.8	0.731
Cervical lordosis C2-C7 (°)	21.0 ± 14.4	21.4 ± 12.5	32.5 ± 12.3	0.000
C7 slope (°)	17.9 ± 10.5	21.3 ± 10.2	28.0 ± 10.4	0.000
T1 slope (°)	22.3 ± 9.6	25.0 ± 10.2	31.8 ± 11.2	0.000
Thoracic kyphosis T1-T12 (°)	40.2 ± 13.0	45.7 ± 12.6	50.6 ± 15.4	0.000
Thoracic kyphosis T5-T12 (°)	30.2 ± 11.1	33.3 ± 10.6	38.5 ± 13.7	0.000
Lumbar lordosis L1-S1 (°)	55.2 ± 13.7	58.2 ± 12.3	53.2 ± 13.0	0.054
Pelvic incidence (°)	47.4 ± 11.4	53.8 ± 14.3	54.4 ± 10.7	0.000
Sacral slope (°)	38.1 ± 10.0	40.3 ± 11.7	35.9 ± 8.3	0.023
Pelvic tilt (°)	9.4 ± 8.6	13.5 ± 7.6	18.5 ± 8.0	0.000
PI-LL L1-S1 (°)	−7.8 ± 11.9	−4.3 ± 10.4	1.3 ± 11.7	0.000
SVA C7 (mm)	9.3 ± 32.7	4.2 ± 32.4	30.9 ± 36.5	0.000
SVA C2 (mm)	25.7 ± 37.1	21.4 ± 36.2	54.7 ± 42.0	0.000
T1 pelvic angle (°)	8.3 ± 6.2	9.7 ± 6.8	15.8 ± 7.1	0.000
Global tilt (°)	10.2 ± 8.3	14.8 ± 7.5	16.5 ± 9.3	0.000
Spino-sacral angle (°)	128.9 ± 10.9	131.6 ± 11.0	123.9 ± 8.2	0.000
Odontoid hip axis (°)	0.9 ± 2.6	1.5 ± 2.8	−0.5 ± 3.8	0.000
T1 tilt (°)	3.2 ± 3.1	4.5 ± 3.0	2.9 ± 4.1	0.010
T9 tilt (°)	8.4 ± 3.6	10.4 ± 3.3	10.7 ± 4.9	0.000

**Table 6:** Comparison of sagittal parameters (mean ± SD) per age in the scoliosis (S) group.

Variable	<20 years	20–50 years	>50 years	p
McGregor slope (°)	−3.2 ± 9.8	4.3 ± 12.8	7.8 ± 23.8	0.005
McGregor C2 angle (°)	12.6 ± 10.2	20.4 ± 12.2	19.4 ± 10.9	0.001
Cervical lordosis C1-C2 (°)	27.3 ± 9.9	29.6 ± 9.4	29.6 ± 9.7	0.403
Cervical lordosis C2-C7 (°)	23.2 ± 15.3	24.9 ± 16.6	33.2 ± 15.1	0.004
C7 slope (°)	18.4 ± 11.3	18.4 ± 10.7	25.5 ± 13.5	0.004
T1 slope (°)	20.7 ± 10.9	22.5 ± 9.2	28.4 ± 13.3	0.002
Thoracic kyphosis T1-T12 (°)	38.7 ± 14.5	45.5 ± 16.0	47.9 ± 11.2	0.003
Thoracic kyphosis T5-T12 (°)	31.5 ± 13.5	38.0 ± 16.9	37.5 ± 10.5	0.035
Lumbar lordosis L1-S1 (°)	55.2 ± 12.7	62.1 ± 15.4	54.3 ± 12.6	0.018
Pelvic incidence (°)	43.8 ± 11.1	49.5 ± 13.5	57.0 ± 11.6	0.000
Sacral slope (°)	38.0 ± 9.2	39.7 ± 10.3	37.8 ± 8.5	0.602
Pelvic tilt (°)	5.8 ± 8.5	9.8 ± 7.6	19.1 ± 11.2	0.000
PI-LL L1-S1 (°)	−11.9 ± 11.8	−12.6 ± 14.1	2.7 ± 15.6	0.000
SVA C7 (mm)	10.9 ± 33.7	−2.8 ± 30.2	31.5 ± 36.9	0.000
SVA C2 (mm)	29.3 ± 39.3	10.9 ± 31.9	48.6 ± 43.3	0.000
T1 pelvic angle (°)	6.7 ± 5.3	7.5 ± 4.7	17.3 ± 9.0	0.000
Global tilt (°)	6.3 ± 8.5	11.9 ± 7.5	16.9 ± 10.8	0.000
Spino-sacral angle (°)	128.4 ± 10.4	131.8 ± 10.9	125.6 ± 9.6	0.022
Odontoid hip axis (°)	0.1 ± 2.8	2.0 ± 2.2	0.1 ± 3.5	0.004
T1 tilt (°)	2.2 ± 3.0	4.4 ± 2.5	2.8 ± 3.9	0.009
T9 tilt (°)	7.1 ± 3.9	10.4 ± 3.6	10.3 ± 5.5	0.000

### 3.5 Correlation between coronal and sagittal alignment parameters

Correlations between coronal and sagittal global alignment parameters were investigated. For each parameter combination, Pearson correlation coefficients R were between −0.2 and 0.2, showing that there was no correlation between coronal and sagittal alignment parameters. When analyzing N- and S- groups separately, there was no correlation in either group.

## 4 Discussion

Despite the considerable literature establishing precise normative values and relations between sagittal alignment parameters, coronal alignment has been overlooked during the last decade. Coronal parameters can be dived into 3 types, describing pelvic, global or shoulder alignment. Most parameters were studied separately without investigating a possible relationship with the sagittal plane. As far as we know, there is no study establishing a comprehensive review of coronal alignment normative values. For some parameters, normative values and ranges are not described in the literature. For example, the Maloney angle is used in neuromuscular scoliosis, and it is associated with a good inter- and intra-observer reliability when measuring pelvic obliquity (Shrader et al., 2018), but no thresholds between normal coronal alignment and spinal deformity are missing. Our results found a normal mean value of 3.5°. Alireza et al. (Moharrami et al., 2023) found a median value of pelvic obliquity of 2° and concluded that a slight pelvic obliquity in healthy population was normal. In our cohort a similar mean value of was 1.7° was found, showing that slight pelvic obliquity can be present in the normal population. When investigating the coronal orientation of caudal lumbar vertebrae, Zhang et al. (2020) found mean values of 3.4° for L4 tilt, which is in line with our findings. This shows that adaptive phenomena exist, which are probably linked to slight pelvic obliquity. Furthermore, Zhang et al. (2020) suggested to assess T1 and pelvic tilt with a mean normal angle of 0.4°. This angle is equivalent to CTPA in our study which accounts for pelvic and T1 plumbline inclination, and which represents an alternative to the widely used C7-CSVL for global coronal alignment. In their study, the mean C7-CSVL distance was 3 mm compared to 8 mm in our normative cohort. Zuckerman et al. (2021) studied the OD-CSVL distance as an alternative to C7-CSVL including the mobile cervical spine. In 67% of their normative cohort, the OD-CSVL distance was <10 mm and in 99% < 30 mm, showing that the coronal plumbline of C2 falls in the middle of the sacrum in most individuals. Clinicians are usually interested in shoulder alignment when examining patients with scoliosis. However, little attention is paid to this parameter in normal populations. Akel et al. (2008) studied coronal shoulder alignment in an adolescent asymptomatic cohort, aiming for a standard as opposed to patients with adolescent idiopathic scoliosis (AIS). They described a mean coracoid angle of 2.2° and a mean coracoid distance of 6.9 mm which is concordant with our findings. This demonstrates that a slight coronal shoulder asymmetry can be considered as normal.

To detect early variations in coronal alignment patterns, we studied mild scoliosis compared to asymptomatic patients. There was no difference in global coronal alignment between mild scoliosis and normal population, except for L4 and L5 tilt. Although statistically significant, differences of 0.3° for CTPA and 0.2 mm for OD-CSVL, we considered below the threshold of radiographic measurement accuracy. Few studies investigated differences between mild scoliosis and asymptomatic subjects in the coronal plane. Langlais et al. (2024) compared mild scoliosis and an asymptomatic group using OD-HA as a bi-planar parameter from three-dimensional EOS reconstructions. In mild scoliosis, 27.5% of coronal OD-HA values were below the 5th or above the 95th percentiles of the normal group. Shu-Man et al. (Han et al., 2023) compared shoulder alignment in mild, moderate and severe scoliosis. Differences between left and right acromioclavicular joint deviations increased from mild to severe scoliosis and varied according to curve types. Schlösser et al. (2021) studied sagittal alignment patterns in mild adolescent idiopathic scoliosis (AIS) with a Cobb angle up to 20° compared to a control group. In mild thoracic scoliosis, 49% were hypho-kyphotic *versus* in 13% thoracolumbar curves and 6% in an asymptomatic group. In our cohort, there was no significant difference between N- and S-groups in the sagittal plane.

When analyzing sagittal alignment parameter variations according to age, it appeared that these apply equally N- and S-groups. Our findings are in line with previous normative data, demonstrating that TK and T1-slope increase with age, whereas LL between L1 and S1 might not change significantly (Sardar et al., 2022; Iyer et al., 2016; Hu et al., 2020). Prost et al. (2023) investigated segmental LL changes in detail on normative radiographs of 1540 subjects and demonstrated that proximal LL between L1 and L4 keeps unchanged, whereas distal LL between L4 and S1 decreases with age. Among pelvic parameters, PT and PI increase with age, whereas SS changes are minor (Sardar et al., 2022; Prost et al., 2023; Bao et al., 2018). Among global sagittal alignment parameters, SVA, TPA and GT are most likely to change as TK increases and the pelvis retroverses with age (Charles et al., 2022a; Iyer et al., 2016). In the cervical segment, CL increases in its caudal segment as TK increases with age (Charles et al., 2022b). McGregor slope and McGregor-C2 angle change as the head slightly extends with age to compensate for increasing CL, thus maintaining horizontal gaze. Similar age-related observations were made in our cohort, but mild scoliosis didn’t influence cervical alignment.

This cross-sectional study has limitations. The evolution of coronal parameters might change over time as scoliosis progresses from moderate to severe deformity. A threshold of 35° was determined for any curve type, but differences could exist depending on primary and secondary curve location. Furthermore, normal subjects and patients with mild scoliosis were included if clinically asymptomatic. However, functional scores, which might influence coronal and sagittal alignment were not assessed. Although we excluded severe radiographic degenerative changes, mild degenerative changes in the lumbar spine could influence coronal alignment and sagittal alignment. These changes were not specifically analyzed. Furthermore, limb length discrepancy was excluded based on radiographic hip and pelvis levels. However, full body EOS radiographs would have been more accurate.

## 5 Conclusion

This study provides a detailed description of coronal alignment parameters and a comprehensive set of normative values in asymptomatic subjects. No significant differences existed in patients with mild scoliosis. Coronal alignment didn’t vary with age, whereas sagittal alignment changed with age. There was no correlation between coronal and sagittal alignment parameters.

## Ethics approval

Institutional review board approval (FC/2019-91) was obtained.

## Funding

This research did not receive any specific grant from funding agencies in the public, commercial, or not-for-profit sectors.

## Declaration of competing interest

Ibrahim Obeid is consultant for Medtronic and Depuy Synthes; he received grants from DePuy Synthes unrelated to this study and royalties from Clariance, Alphatec and Spineart. Louis Boissière is consultant for Neo and Euros; he received grants from DePuy Synthes unrelated to this study. Yann Philippe Charles is consultant for Stryker, Clariance, Spinevision and Ceraver; he received royalties and grants unrelated to this study from Stryker and Clariance. Ahmed Gharbi, Daniel Larrieu, Maxime Huneidi, Pablo Lamotte-Paulet, Mekki Tamir and Carlos Aleman have no conflict of interest.
